# Combined Exposure to Ragweed and House Dust Mite Exacerbates Airway Epithelial Barrier Dysfunction: A Multimodal Approach

**DOI:** 10.3390/medicina62050980

**Published:** 2026-05-17

**Authors:** Elena-Larisa Zimbru, Răzvan-Ionuț Zimbru, Manuela Grijincu, Florina-Maria Bojin, Maria-Roxana Buzan, Sorin Dan Chiriac, Gabriela Tănasie, Laura Haidar, Crenguta Livia Calma, Carmen Panaitescu

**Affiliations:** 1 Center of Immuno-Physiology and Biotechnologies, Department of Functional Sciences, “Victor Babes” University of Medicine and Pharmacy, 300041 Timisoara, Romania; elena.zimbru@umft.ro (E.-L.Z.);; 2 Research Center for Gene and Cellular Therapies in the Treatment of Cancer—OncoGen, Timis County Emergency Clinical Hospital “Pius Brinzeu”, 300723 Timisoara, Romania; 3 Discipline of Surgery III, Department X, Faculty of Medicine, “Victor Babes” University of Medicine and Pharmacy, 300041 Timisoara, Romania

**Keywords:** airway epithelial barrier dysfunction, allergen co-exposure, house dust mites, ragweed pollen, tight junctions, multimodal analysis, epithelial integrity, allergic asthma

## Abstract

*Background and Objectives:* Dysfunction of the airway epithelial barrier is increasingly recognized as an early pathogenic mechanism in allergic respiratory diseases. Although individual aeroallergens such as ragweed (RW) pollen and house dust mite (HDM) are known to impair epithelial integrity, the effects of combined exposure, more reflective of real-world conditions, remain insufficiently characterized. This study aimed to evaluate the impact of single versus combined allergen exposure on airway epithelial barrier function using a multimodal experimental approach. *Materials and Methods:* Differentiated normal human bronchial epithelial (NHBE) cells were exposed to RW (100 µg/mL), HDM (100 µg/mL), or a combined extract (RW + HDM; total 100 µg/mL). Barrier function under air–liquid interface conditions was assessed by transepithelial electrical resistance (TEER), while real-time cellular responses were evaluated using xCELLigence impedance monitoring. Structural alterations were examined by occludin-based immunofluorescence imaging, and transcriptional changes associated with epithelial stress and inflammation were analyzed by RT-qPCR. *Results:* Allergen exposure induced time- and concentration-dependent impairment of epithelial barrier function. Combined exposure resulted in the most pronounced and sustained reduction in TEER and impedance measurements. These functional changes were accompanied by disruption of tight junction organization and coordinated transcriptional modulation of genes involved in inflammatory and stress responses. *Conclusions:* Combined exposure to RW and HDM extracts induced more severe and persistent epithelial barrier dysfunction than individual allergens. These findings support the role of the airway epithelium as a central regulator of allergic airway disease and highlight barrier disruption as an early pathogenic event. The multimodal framework applied in this study provides an integrated platform for investigating epithelial responses to complex environmental exposures.

## 1. Introduction

The airway epithelium constitutes the first line of defense against inhaled environmental agents, forming both a physical and immunological barrier that is essential for maintaining respiratory homeostasis [1,2]. In healthy airways, epithelial cells are tightly connected through intercellular junctional complexes and actively participate in innate immune responses, mucociliary clearance and tissue repair [3,4,5,6]. Disruption of epithelial barrier integrity has been increasingly recognized as a central event in the pathogenesis of allergic airway diseases, including asthma and allergic rhinitis, where enhanced permeability facilitates allergen penetration and amplifies inflammatory signaling cascades [7,8].

**Figure 1:**
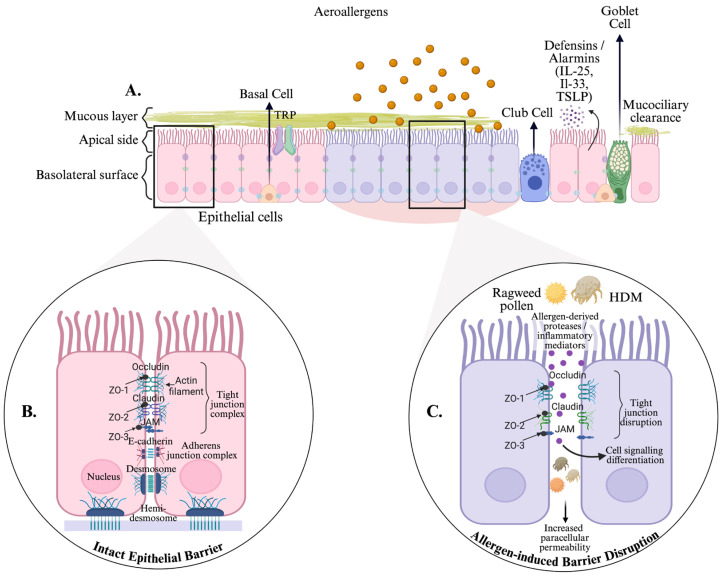
Allergen-induced airway epithelial barrier dysfunction. (**A**) Schematic overview of the airway epithelial barrier showing the organization of epithelial cell populations, including basal, club and goblet cells, covered by a protective mucus layer that contributes to mucociliary clearance and innate immune defense. (**B**) Intact epithelial barrier under physiological conditions. Well-organized intercellular junctional complexes—including tight junction proteins (occludin, claudins, junctional adhesion molecules and ZO proteins) and adherens junctions (E-cadherin)—maintain epithelial polarity and restrict paracellular permeability. (**C**) Allergen-induced epithelial barrier disruption. Exposure to aeroallergens such as ragweed pollen and house dust mite (HDM) and their associated proteases and inflammatory mediators disrupts tight junction organization, increases paracellular permeability and facilitates allergen penetration across the epithelial layer. This process is accompanied by epithelial signaling and the release of antimicrobial peptides (defensins) and epithelial alarmins (IL-25, Il-33 and TSLP). This is a schematic representation based on current knowledge. Abbreviations: ZO-1, ZO-2, ZO-3—zonula occludens-1, -2, -3; JAM—junctional adhesion molecule; HDM—house dust mites; IL-25—interleukin-25; Il-33—interleukin-33; TSLP—thymic stromal lymphopoietin; TRP—transient receptor potential channels. Created with BioRender.com.

Respiratory allergens, including house dust mite (HDM) and ragweed (RW) pollen, possess intrinsic proteolytic activity or induce oxidative and inflammatory stress, enabling them to directly compromise epithelial junctions [9,10,11,12,13]. These effects include alterations in tight junction proteins, cytoskeletal reorganization and impaired barrier function (Figure 1) [10,14,15,16]. Despite extensive evidence linking allergen exposure to epithelial dysfunction, the mechanisms by which different allergens influence epithelial barrier integrity and cellular behavior remain incompletely characterized [7,17,18].

Beyond classical IgE-mediated sensitization, increasing evidence indicates that allergic respiratory diseases also involve complex non-IgE-mediated mechanisms, including epithelial-derived alarmin signaling, innate immune activation, leukocyte-mediated inflammatory responses and dysregulated epithelial–immune cross-talk [19,20,21]. Recent advances in precision medicine and disease endotyping have emphasized that clinically similar allergic phenotypes may arise from distinct underlying molecular and immunological pathways [19]. In this context, airway epithelial dysfunction is increasingly recognized not only as a structural abnormality, but also as an active immunoregulatory process capable of amplifying both innate and adaptive inflammatory responses [22]. Emerging investigations have increasingly emphasized the importance of exploring cellular and humoral immunoreactivity beyond traditional IgE-focused frameworks, particularly in patients presenting clinically relevant allergic manifestations despite negative or inconclusive IgE-based assessments [19,21]. Experimental approaches capable of integrating functional, structural and molecular alterations may, therefore, contribute to a broader characterization of allergen-induced epithelial responses beyond conventional IgE-centered paradigms and support the development of more individualized therapeutic strategies in allergic respiratory disease.

Normal human bronchial epithelial (NHBE) cells provide a physiologically relevant in vitro model for investigating airway epithelial responses to environmental stimuli [18,23,24]. When exposed to respiratory allergens, NHBE cells exhibit changes in barrier properties, inflammatory mediator release and cellular morphology that mirror key aspects of airway disease pathology [18]. Although airway epithelial dysfunction has been widely implicated in allergic airway disease, most previous studies have relied on single readout systems assessing barrier integrity, structural alterations or molecular responses independently. However, reliance on single experimental readouts may fail to capture the complexity of epithelial barrier disruption, emphasizing the need for integrated analytical approaches [1]. Recent advances in allergic airway disease research increasingly support the concept that airway epithelial dysfunction represents not only a structural barrier defect, but also an active immunoregulatory process involved in innate immune activation, epithelial–immune cross-talk and amplification of inflammatory signaling pathways [6,25,26]. Several recent studies investigating airway epithelial dysfunction in allergic airway disease have predominantly focused on isolated analytical modalities, including functional barrier assessment by transepithelial electrical resistance (TEER), structural evaluation of tight junction organization by immunofluorescence imaging or targeted transcriptional analyses of inflammatory and remodeling-associated genes [7,27,28,29]. While these approaches have substantially advanced the understanding of epithelial barrier pathology, single-modality investigations may only partially capture the complex and dynamic nature of epithelial responses to environmental allergens. Current experimental and translational approaches increasingly emphasize the importance of integrating functional, structural and molecular analyses to more comprehensively characterize epithelial responses to environmental allergens and better understand disease heterogeneity, airway remodeling and therapeutic responsiveness [2,20]. In parallel, allergen-specific immunotherapy and experimental desensitization models further support the importance of epithelial barrier biology in the modulation of allergic airway disease [25].

**Figure 2:**
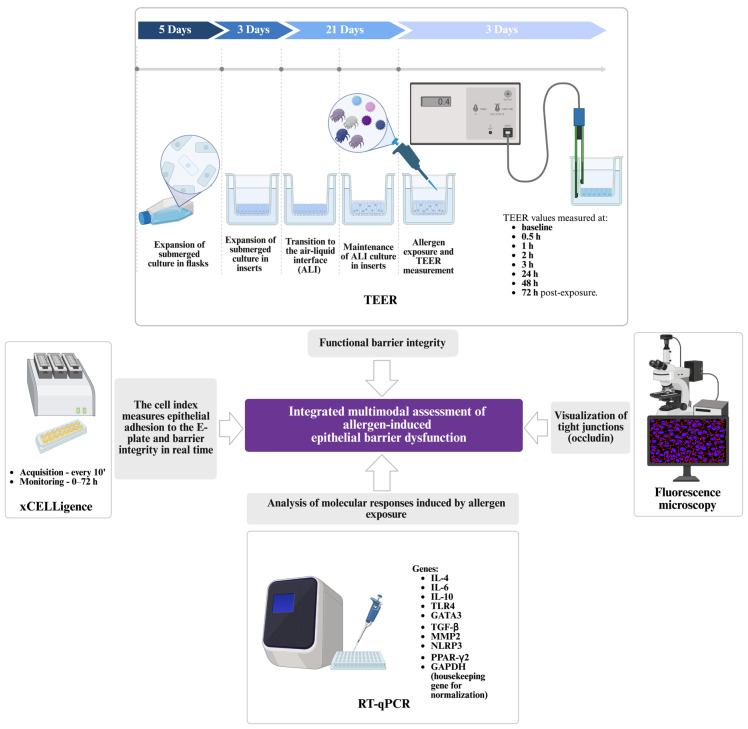
Experimental design. Transepithelial electrical resistance (TEER) measurements, real-time cellular impedance analysis (xCELLigence) were used to evaluate epithelial barrier function in NHBE cells under various experimental conditions. TEER values were recorded at baseline (prior to allergen exposure), at 30 min post-exposure, at 1, 2, 3 and 4 h, and then daily for an additional three days to assess both acute and sustained changes in barrier integrity. xCELLigence monitoring provided continuous, label-free measurements of cellular impedance, reflecting changes in cell adhesion and barrier-related behavior over time. Immunofluorescence microscopy was performed to visualize tight junction organization, with occludin stained in red and nuclei counterstained with DAPI (blue). RT-qPCR analysis was conducted to quantify molecular expression changes associated with epithelial barrier function and cellular responses. Abbreviations: TEER: transepithelial electrical resistance; ALI: air–liquid interface; RT-qPCR: reverse transcription quantitative polymerase chain reaction; IL-4: interleukin-4; IL-6: interleukin-6; IL-10: interleukin-10; TLR4: toll-like receptor 4; GATA3: GATA-binding protein 3; TGF-β: transforming growth factor beta; MMP2: matrix metalloproteinase-2; NLRP3: NLR family pyrin domain containing 3; PPAR-γ2: peroxisome proliferator-activated receptor gamma 2; GAPDH: glyceraldehyde-3-phosphate dehydrogenase; h: hours; ′: minutes. Created with BioRender.com.

The present study provides an integrated multimodal framework combining functional barrier assessment, structural characterization and transcriptional profiling in a unified experimental design. Transepithelial electrical resistance (TEER) measurements and real-time cellular impedance analysis (xCELLigence) were used to evaluate epithelial barrier function under various experimental conditions. Immunofluorescence microscopy was performed to visualize tight junction architecture. Finally, RT-qPCR analysis was conducted to quantify gene expression changes associated with epithelial barrier function and cellular responses to allergen exposure. Figure 2 summarizes the experimental workflow, illustrating NHBE cells following allergen exposure during a 72-h monitoring period. The figure integrates functional assessment of epithelial barrier integrity by TEER, occludin-based immunofluorescence imaging, real-time Cell Index measurements using the xCELLigence system and transcriptional profiling of immune-related genes.

By integrating functional, structural and molecular readouts, this multimodal approach enables a comprehensive characterization of allergen-induced epithelial dysfunction and provides greater insight into how individual and combined respiratory allergens impair airway epithelial integrity. These findings contribute to a more refined understanding of airway barrier pathology in allergic respiratory disease and support the development of mechanism-based therapeutic strategies. Airway epithelial barrier disruption may represent an early pathogenic event in allergic asthma, promoting sustained inflammatory responses that extend beyond the local airway environment and may contribute to systemic inflammation with potential extra-pulmonary, including vascular, consequences [30,31].

## 2. Materials and Methods

### 2.1. Preparation of Purified Allergen Extracts

The study used extracts of ragweed pollen (*Ambrosia artemisiifolia*; RW) and house dust mites (*Dermatophagoides pteronyssinus*; HDM), both sourced from Allergon AB (Thermo Fisher Scientific Inc., Ängelholm, Sweden). The HDM extract was prepared using 0.3 g suspensions *Dermatophagoides pteronyssinus* in 5 mL of DPBS (Gibco, Thermo Fisher Scientific, Waltham, MA, USA) supplemented with a protease inhibitor mixture (SigmaFAST, Sigma Aldrich, St. Louis, MO, USA). After homogenization, the suspension was gently vortexed overnight at 4 °C (20 rpm) and then centrifuged at 18,000× *g* for 20 min at 4 °C. The supernatant was dialyzed against PBS and sterilized through a 0.22 μm filter. The ragweed pollen extract was prepared from *Ambrosia artemisiifolia* pollen following the extraction procedures as previously established [15,18,32,33,34]. The final protein concentration of the prepared extracts was 5 mg/mL for ragweed pollen and 4 mg/mL for the HDM preparation, quantified using the BCA protein assay (Thermo Fisher Scientific, Waltham, MA, USA).

Cell cultures were then exposed to single-allergen exposures RW (100 µg/mL), HDM (100 µg/mL), as well as combined HDM and RW pollen extract mixture HR100 corresponding to a combined exposure of 50 µg/mL HDM + 50 µg/mL RW (total 100 µg/mL), adding concentrations of 10 and 200 µg/mL for each for the xCELLigence real-time cell analysis.

### 2.2. Differentiating NHBE Cells into a Bronchial Epithelium Using an Air-Liquid Interface System

NHBE cells (Bronchial Epi Cells for B-ALI, Lonza Walkersville Inc., Walkersville, MD, USA) were employed to establish a differentiated in vitro model of the human airway epithelium. This system reproduces the physiological morphological and functional properties of bronchial epithelium, including epithelial barrier formation, apical microvilli, coordinated ion transport and intact tight junction complexes. Cell culture procedures were conducted according to the manufacturer’s recommendations, with minor methodological adaptations based on previously published protocols.

NHBE cells were first expanded in T75 tissue culture flasks (Corning, New York, NY, USA) at 37 °C in a humidified incubator with 5% CO_2_. The PneumaCult™-ALI medium (STEMCELL Technologies, Vancouver, BC, Canada) used in cell cultures was supplemented with the required growth factors. Once cultures reached approximately 70–90% confluence, cells were detached, counted and transferred to 12-well Transwell inserts (Costar^®^, Corning, New York, NY, USA) with 0.4 µm pore polyester membranes. Prior to seeding, the inserts were coated with 100 µL fibronectin (20 µg/mL, Gibco, Thermo Fisher Scientific, Waltham, MA, USA) to facilitate attachment. Cells were cultured at a density of 1000 cells/mm^2^.

Differentiation was achieved through a structured, three-step process that extended over a period of 24 days. In the initial phase (days 0–3), the cells were immersed to promote the formation of a single layer. The cultures were then transferred to an ALI, with medium being supplied exclusively to the basolateral compartment, thus allowing the apical surface to be exposed to air and mimicking the airway physiology in vivo. Over the following weeks, NHBE cells progressively developed a pseudostratified pattern with ciliated and secretory cell phenotypes. The basolateral medium was refreshed every 48 h, while the apical surface remained uncovered. Starting in the second week, accumulated mucus was carefully removed as needed with PBS washes to support optimal differentiation.

Triton X-100 (Thermo Fisher Scientific, Waltham, MA, USA) was used as a positive control to induce membrane disruption and confirm loss of viability. In parallel, real-time impedance monitoring using the xCELLigence system enabled continuous assessment of cell integrity and adherence, serving as an indirect indicator of cell viability. The absence of a comparable collapse in cell index values in allergen-treated groups suggests that the observed changes primarily reflect functional epithelial responses rather than overt cytotoxicity.

Throughout the ALI culture period, epithelial morphology, confluence and barrier maturation were assessed regularly to confirm the establishment of a functionally competent bronchial epithelium.

### 2.3. Real-Time Assessment of Epithelial Barrier Integrity Using xCELLigence System

Barrier integrity of NHBE cells was continuously assessed using the xCELLigence Real-Time Cell Analysis dual purpose (RTCA DP) system (ACEA Biosciences Inc., San Diego, CA, USA) connected to RTCA software 1.2.1, which enables non-invasive, label-free impedance monitoring. Aliquots of 200 μL of cell suspension, at a concentration of 1.5 × 10^5^ were seeded into fibronectin-coated 16-well E-Plates at a density adapted to reach confluence within 24 h, ensuring the establishment of a stable epithelial layer. Electrical impedance was recorded automatically every 10 min, generating a Cell Index, a unit that reflects cell adhesion, morphology, barrier function and epithelial integrity. Once cultures reached the plateau phase, corresponding to stable Cell Index values, the cells were exposed to the designated allergen preparations, as mentioned in Section 2.1. The last impedance value prior to exposure was set as the normalization point and subsequent measurements were expressed as Normalized Cell Index to track dynamic barrier responses over time. This approach, as previously described for respiratory epithelial barrier analysis, provides high temporal resolution and increased sensitivity for detecting subtle allergen-induced impairments compared with endpoint-based measurements. To obtain an integrated assessment of epithelial barrier dynamics over time, the AUC was calculated from normalized cell index values over the 72-h monitoring period.

### 2.4. Transepithelial Electrical Resistance (TEER) Measurement for the Assessment of Epithelial Barrier Integrity and Allergen-Induced Responses

TEER measurements were performed using an Epithelial Voltohmmeter (EVOM2; World Precision Instruments, Sarasota, FL, USA) in a Transwell culture system. Electrodes were positioned in the apical and basolateral compartments to quantify the electrical resistance across the epithelial monolayer, providing an index of paracellular permeability and barrier integrity. A low-voltage alternating current was applied and resistance values were corrected for membrane surface area and expressed as Ω·cm^2^ [35].

Differentiated bronchial epithelial cultures were exposed to HDM extract (100 µg/mL), RW pollen extract (100 µg/mL), or their combination HR100 corresponding to a combined exposure of 50 µg/mL HDM + 50 µg/mL RW (total 100 µg/mL). TEER values were recorded before allergen exposure to establish baseline levels, followed by measurements at 30 min, hourly during the first 4 h and subsequently at 24, 48 and 72 h. This sampling strategy enabled the assessment of both early and delayed alterations in barrier function. Also, the AUC was calculated from normalized TEER values over the 72-h time course to provide an integrated measure of epithelial barrier integrity.

Each experimental condition was evaluated in triplicate and repeated in three independent runs using cells derived from the same batch. Baseline TEER values were used for normalization and results are presented as mean ± SEM.

### 2.5. Assessment of Tight Junction Integrity Using Immunofluorescence with Occludin

The immunofluorescence staining was used to assess the distribution of occludin, a major component of tight junction complexes. NHBE cells were cultured on 4-well chamber slides (Nunc™ Lab-Tek; Thermo Fisher Scientific, Waltham, MA, USA) that had been precoated with 200 µL of fibronectin (20 µg/mL) to support optimal adhesion. Each well received approximately 25,000 cells, which were allowed to attach and proliferate for 48 h in complete PneumaCult™-ALI basal medium. The medium was then replaced with PneumaCult™-ALI maintenance medium and the cultures were incubated for a further 72 h to support early differentiation.

The cells were then exposed for 72 h to the allergen preparations and combinations described in Section 2.1. After treatment, the cultures were fixed with cooled methanol (−20 °C, 10 min, Thermo Fisher Scientific, Waltham, MA, USA), rinsed in PBS and incubated in 1% bovine serum albumin (Thermo Fisher Scientific, Waltham, MA, USA) for 3 h to minimize nonspecific antibody binding. Immunolabeling was performed overnight at 4 °C using an anti-occludin monoclonal antibody (OC-3F10) directly conjugated to Alexa Fluor 594 (Invitrogen, Thermo Fisher Scientific, Waltham, MA, USA). Nuclear counterstaining was performed using DAPI formulated in SlowFade™ Gold antifade reagent (Invitrogen, Thermo Fisher Scientific, Waltham, MA, USA).

Fluorescent images were captured using the EVOS™ FL Auto 2 imaging system (Thermo Fisher Scientific Inc., Bothell, WA, USA). Image analysis and signal quantification were performed in ImageJ.JS (version 1.53m). 

### 2.6. Gene Expression Analysis by Quantitative Real-Time PCR

#### 2.6.1. RNA Isolation from Differentiated NHBE Cells

NHBE cells were cultured and differentiated to form a pseudostratified bronchial epithelium on ibidi slides for 7 days. Afterwards, the cells were exposed to the specific allergen preparations for a period of 72 h, as described in Section 2.1. At the end of the exposure period, cells were harvested from Transwell inserts by gentle trypsin treatment. Total RNA was isolated using the GenElute™ Mammalian Total RNA Miniprep Kit (Sigma-Aldrich, St. Louis, MO, USA), according to the manufacturer’s protocol. Following extraction, RNA samples were stabilized in RNAlater solution (Thermo Fisher Scientific, Waltham, MA, USA) and stored at −80 °C until further processing. RNA concentration and purity were evaluated by fluorometry and RNA yield was quantified using a Qubit™ 4 fluorometer (Thermo Fisher Scientific, Waltham, MA, USA) prior to further processing.

#### 2.6.2. cDNA Synthesis

First-strand complementary DNA was synthesized from total RNA using the SuperScript™ VILO™ cDNA Synthesis Kit (Invitrogen, Thermo Fisher Scientific, Carlsbad, CA, USA). For each reaction, up to 1 µg of total RNA was reverse-transcribed in a final volume of 20 µL, following the manufacturer’s instructions. The reverse transcription reaction consisted of incubation at 25 °C for 10 min, 42 °C for 60 min and enzyme inactivation at 85 °C for 5 min. The resulting cDNA was stored at −20 °C until use in quantitative PCR assays.

#### 2.6.3. Quantitative Real-Time PCR

RT-qPCR was performed using SYBR™ Green technique with the Applied Biosystems™ SYBR™ Green PCR Master Mix (Thermo Fisher Scientific, Waltham, MA, USA). Amplification reactions were carried out on a LightCycler^®^ 480 II Real-Time PCR System (Roche Diagnostics, Mannheim, Germany), using LightCycler^®^ 480 Software version 1.5.1. Each reaction was prepared in a final volume of 20 µL, containing SYBR Green Master Mix, gene-specific primers and cDNA template.

Gene expression analysis targeted genes involved in immune regulation, innate immune signaling and airway remodeling that are relevant to asthma pathophysiology. The genes included are IL-4, IL-6, IL-10, GATA3, TLR4, NLRP3, TGF-β, PPAR-γ2 and MMP2, with GAPDH (Biozyme, Cluj-Napoca, Romania) used as a housekeeping gene for normalization, based on previous validation studies demonstrating stable expression in human epithelial cell lines (Table 1) [36,37]. All reactions were run in duplicates and non-template controls were included to exclude contamination. Relative gene expression levels were calculated using the comparative Ct (2^−ΔΔCt^) method.

### 2.7. Statistical Analysis

All experiments were performed in at least three independent biological replicates unless otherwise specified. Data are presented as mean ± SEM. Statistical analyses and graphical representations were performed using GraphPad Prism version 10.6.1 (GraphPad Software, Boston, MA, USA).

For multiple group comparisons, one-way or two-way ANOVA was used as appropriate, followed by Tukey’s or Dunnett’s post hoc test.

To explore potential interaction effects between RW and HDM exposure, exploratory two-way ANOVA interaction analyses were additionally performed for TEER and impedance-derived AUC values, using RW and HDM exposure as independent factors.

For RT-qPCR experiments, statistical analyses were performed on ΔCt values prior to fold-change calculation using the 2^−ΔΔCt^ method. Amplification specificity was verified by melt curve analysis. A *p*-value < 0.05 was considered statistically significant.

### 2.8. Ethical Approval

This study was approved by the Ethics Committee of “Victor Babes” University of Medicine and Pharmacy in Timisoara, Romania (protocol code no.40/20.12.2023 S 2026).

## 3. Results

### 3.1. Functional Assessment of Epithelial Barrier Integrity

#### 3.1.1. xCELLigence-Based Analysis Following Allergen Exposure

**Figure 3:**
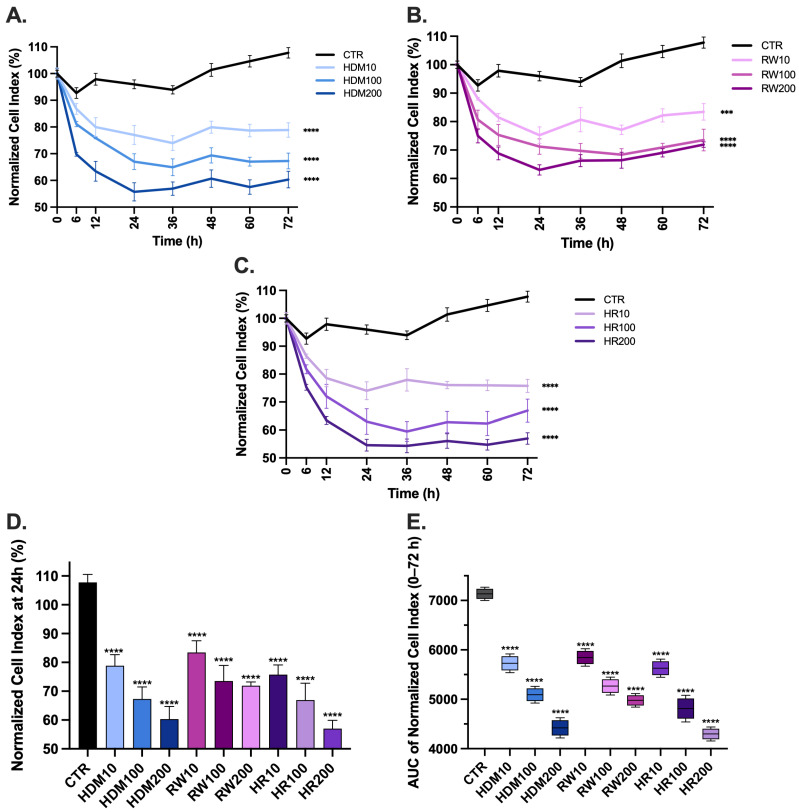
Time-dependent effects of single and combined allergen exposure on bronchial epithelial barrier function assessed by real-time impedance analysis. Normalized cell index (%) of NHBE cells monitored using the xCELLigence real-time cell analysis system over 72 h following exposure to HDM, RW pollen or combined HDM + RW extracts at concentrations of 10, 100 and 200 µg/mL. Cell index values were normalized to baseline and expressed as percentage of baseline. (**A**–**C**) Time-course of normalized cell index following: (**A**) exposure to HDM compared to control, (**B**) exposure to RW compared to control, or (**C**) exposure to combined HDM + RW (HR) compared to control (**D**), normalized cell index at 24 h, enabling direct comparison of allergen- and dose-dependent effects. (**E**) Area under the curve (AUC) of normalized cell index over the 0–72 h interval. Measurements were recorded at 0, 6, 12, 24, 36, 48, 60, and 72 h post-exposure. Data are presented as mean ± SEM from three independent experiments performed in triplicate. Statistical analysis significance for time-course data (**A**–**C**) was conducted using two-way ANOVA followed by Tukey’s multiple comparisons test. For the 24 h endpoint (**D**) and AUC values (**E**), comparisons were performed using one-way ANOVA followed by Dunnett’s multiple comparisons test versus control. Statistical significance is indicated in the graphs (*** *p* < 0.001, **** *p* < 0.0001). The groups are labeled as follows: CTR: control; RW100: RW extract 100 µg/mL; HDM100: HDM extract 100 µg/mL; HR100: combined exposure of 50 µg/mL HDM + 50 µg/mL RW (total 100 µg/mL).

Real-time impedance monitoring using the xCELLigence system revealed pronounced time-dependent alterations in epithelial barrier integrity following allergen exposure over the 0–72 h observation period (Figure 3). Control cultures maintained stable normalized Cell Index values throughout the experiment, consistent with preserved epithelial adhesion and barrier-related behavior.

Exposure to HDM extract caused a rapid decrease in Cell Index values during the initial phase of monitoring, followed by partial stabilization at later time points (Figure 3A). The magnitude of this reduction increased in a concentration-dependent manner. At 24 h, HDM exposure significantly reduced Cell Index values to 77.01 ± 3.57% for HDM10, 67 ± 2.97% for HDM100 and 55.74 ± 3.40% for HDM200 (*p* < 0.0001 for all conditions vs. control). These findings indicate a dose-dependent impairment of epithelial cell adhesion and barrier integrity, with the highest concentration producing the most pronounced and sustained decrease in impedance.

RW pollen exposure produced a pattern of epithelial impairment similar to that observed with HDM, characterized by an early reduction in Cell Index values followed by partial recovery. Nevertheless, values remained significantly lower than those of control cultures throughout the monitoring period (Figure 3B). At 24 h, RW treatment decreased Cell Index values to 75.20 ± 2.90% for RW10, 71.23 ± 2.72% for RW100 and 64.02 ± 1.81% for RW200 (*p* < 0.0001 for RW100 and RW200 vs. control, *p* < 0.001 for RW10). Although the magnitude of reduction was less pronounced than that observed for HDM exposure, RW exposure induced sustained impairment of epithelial integrity compared with untreated controls.

Combined allergen exposure (HDM + RW; HR) resulted in the greatest reduction in epithelial barrier function (Figure 3C). HR-treated cultures exhibited a marked and sustained decrease in Cell Index values across all time points compared with both control and single-allergen conditions. At 24 h, Cell Index values decreased to 74.02 ± 3.21% for HR10, 63.01 ± 4.56% for HR100 and 54.57 ± 2.12% for HR200 (*p* < 0.0001 for all conditions vs. control).

Quantitative analysis of epithelial barrier integrity at 24 h (Figure 3D) demonstrated a significant reduction in normalized cell index across all allergen-exposed groups compared with control (*p* < 0.0001), supporting early impairment of epithelial barrier function. A clear dose-dependent effect was observed, with higher allergen concentrations inducing more pronounced reductions. Notably, combined exposure to RW and HDM resulted in lower values compared with individual allergen treatments, supporting an enhanced disruptive effect of co-exposure on epithelial integrity.

To evaluate cumulative epithelial responses, the area under the curve (AUC) was calculated over the 0–72 h interval using the trapezoidal rule. Control cultures showed the highest cumulative Cell Index values (CTR: 7134 ± 68.74), consistent with preserved epithelial integrity. HDM exposure induced a significant dose-dependent decrease in AUC values, reaching 5728 ± 97.14 (19.71% reduction vs. control), 5092 ± 86.41 (28.62% reduction) and 4422 ± 103.80 (38.01% reduction) for HDM10, HDM100 and HDM200, respectively.

Similarly, RW exposure resulted in reduced cumulative AUC values compared with controls, with decreases of approximately 18.05% (RW10: 5846 ± 90.10), 26.17% (RW100: 5267 ± 92.61) and 30.22% (RW200: 4978 ± 70.29), indicating persistent impairment of epithelial barrier function.

Combined allergen exposure produced the most pronounced cumulative effect, with AUC values reduced to 5628 ± 94.16 (21.11% reduction vs. control), 4812 ± 137.7 (32.55% reduction) and 4298 ± 71.90 (39.75% reduction) for HR10, HR100 and HR200, respectively. AUC analysis confirmed a significant reduction in cell index across all allergen-treated groups compared to control (*p* < 0.0001), with a clear dose-dependent effect and the most pronounced impairment observed under combined allergen exposure.

Two-way ANOVA of impedance-derived AUC values revealed a significant interaction between RW100 and HDM100 exposure (*p* < 0.0001), supporting the presence of a synergistic-like interaction under co-exposure conditions.

#### 3.1.2. Transepithelial Electrical Resistance Monitoring of Allergen-Induced Barrier Dysfunction

**Figure 4:**
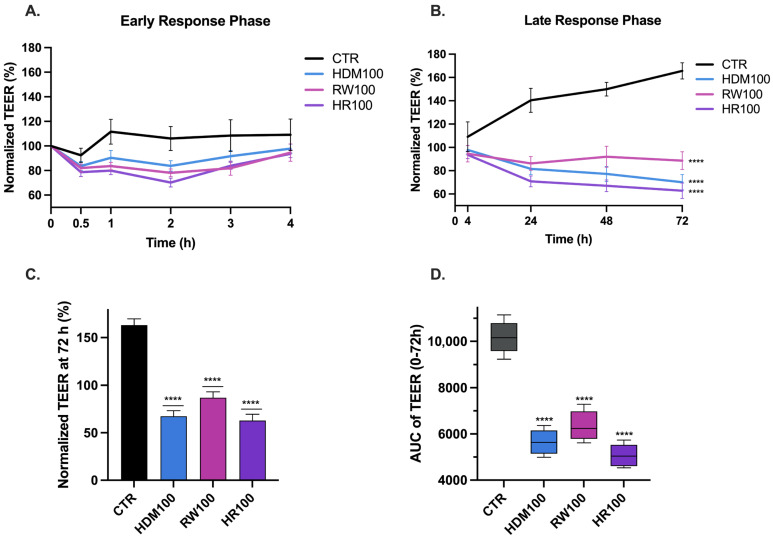
Time-dependent effects of allergen exposure on epithelial barrier integrity assessed by transepithelial electrical resistance. TEER measurements were used to evaluate epithelial barrier integrity in bronchial epithelial cells following exposure to allergen extracts. TEER values were normalized to baseline for each replicate and expressed as percentage of baseline. (**A**) Early response phase (0–4 h): Time-course of normalized TEER following exposure to HDM, RW or combined HDM + RW (HR). (**B**) Late response phase (4–72 h): Time-course of normalized TEER over the extended observation period (4, 24, 48 and 72 h). Statistical analysis significance for time-course data (**A**,**B**) was conducted using two-way ANOVA followed by Tukey’s multiple comparisons test. (**C**) Normalized TEER at 72 h, enabling comparison across treatment groups. (**D**) Area under the curve (AUC) of TEER over the 0–72 h interval. Statistical analysis significance was performed using one-way ANOVA followed by Dunnett’s multiple comparisons test versus control. Statistical significance is indicated in the graphs, compared to CTR (**** *p* < 0.0001). The groups are labeled as follows: CTR: control; RW100: RW extract 100 µg/mL; HDM100: HDM extract 100 µg/mL; HR100: combined exposure of 50 µg/mL HDM + 50 µg/mL RW (total 100 µg/mL).

TEER measurements demonstrated dynamic changes in epithelial barrier integrity following allergen exposure (Figure 4). Across all experimental conditions, TEER values showed a transient increase during the early phase after exposure (0–4 h) (Figure 4A), followed by a progressive decline that persisted throughout the observation period (4–72 h) (Figure 4B). At 72 h, allergen-exposed groups exhibited a marked reduction in TEER compared to control (Figure 4C), supporting sustained epithelial barrier impairment.

During the early response phase (0–4 h), allergen-exposed cultures showed an initial elevation in TEER relative to baseline, followed by a progressive decline during the subsequent 24 h. During the late phase of monitoring (24–72 h), TEER values progressively declined and remained persistently reduced, consistent with persistent compromise of epithelial barrier integrity. Single-allergen exposure induced progressive time-dependent reductions, with HDM100 and RW100 decreasing TEER values at 68.47 ± 5.96% and 88.61 ± 7.58%, respectively, relative to baseline at 72 h. In contrast, combined allergen exposure resulted in a larger and more sustained decrease in TEER, with HR100 producing a reduction to 62.86 ± 6.58% compared with baseline values (*p* < 0.0001 for all conditions vs. control). When compared with single allergen exposures, the effect of combined allergen exposure reached statistical significance only relative to RW100 (*p* = 0.0489).

The magnitude and persistence of TEER reduction were dependent on allergen composition, with combined exposure consistently producing the greatest loss of barrier integrity throughout the monitoring period.

To assess cumulative effects, AUC values were calculated as described above. Control cultures exhibited the highest cumulative TEER values (AUC = 10,189 ± 487.0), consistent with preserved epithelial barrier integrity. Exposure to individual allergens significantly reduced cumulative resistance values, with HDM100 and RW100 yielding AUC values of 5783 ± 296.80 and 6450 ± 424.40, corresponding to reductions of 43.5% and 36.7% relative to control, respectively (*p* < 0.0001 for both vs. control). Combined allergen exposure produced the most pronounced cumulative reduction, with HR100 yielding an AUC of 5182 ± 281.30, representing a 49.1% decrease compared with control (*p* < 0.0001 vs. control).

Two-way ANOVA revealed a significant interaction between the RW and HDM exposures (*p* < 0.0001), indicating that the combined effect on epithelial barrier function is suggesting a synergistic-like or a greater-than-additive interaction.

### 3.2. Structural Alterations of Tight Junctions Revealed by Occludin Immunofluorescence

**Figure 5:**
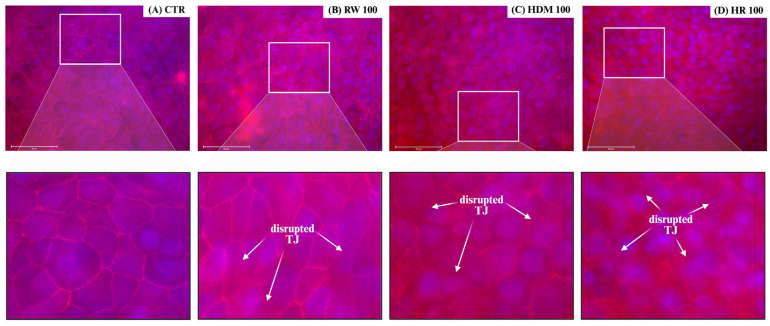
Immunofluorescence staining of occludin in NHBE cell cultures. NHBE cells were exposed to allergen extracts to evaluate tight junction (TJ) alterations induced by allergen exposure. Fixed cells were stained for occludin (red), while cell nuclei were counterstained with DAPI (blue), and images were acquired using fluorescence microscopy. (**Upper**) panels show overview images and (**lower**) panels represent magnified regions of interest. Arrows indicate areas of TJ disruption. Scale bar: 50 μm. Experimental groups were defined as follows: (**A**) CTR, control; (**B**) RW100, ragweed pollen extract (100 μg/mL); (**C**) HDM100, house dust mite extract (100 μg/mL); (**D**) HR100, combined exposure of 50 µg/mL HDM + 50 µg/mL RW (total 100 µg/mL).

Immunofluorescence staining of occludin revealed marked allergen-induced alterations in epithelial tight junction organization (Figure 5). In control NHBE cultures, occludin displayed a continuous and linear distribution along cell-cell borders, concordant with intact junctional architecture and preserved epithelial organization.

Exposure to allergen extracts resulted in progressive structural changes, including fragmentation and discontinuity of occludin localization, with focal loss of junctional staining. These alterations were most pronounced in cultures exposed to combined RW and HDM extracts (HR100), which exhibited irregular occludin distribution, widened intercellular spaces and marked loss of junctional continuity. In contrast, single-allergen exposure (RW100 or HDM100) induced less extensive structural alterations, characterized primarily by localized junctional disruption.

**Figure 6:**
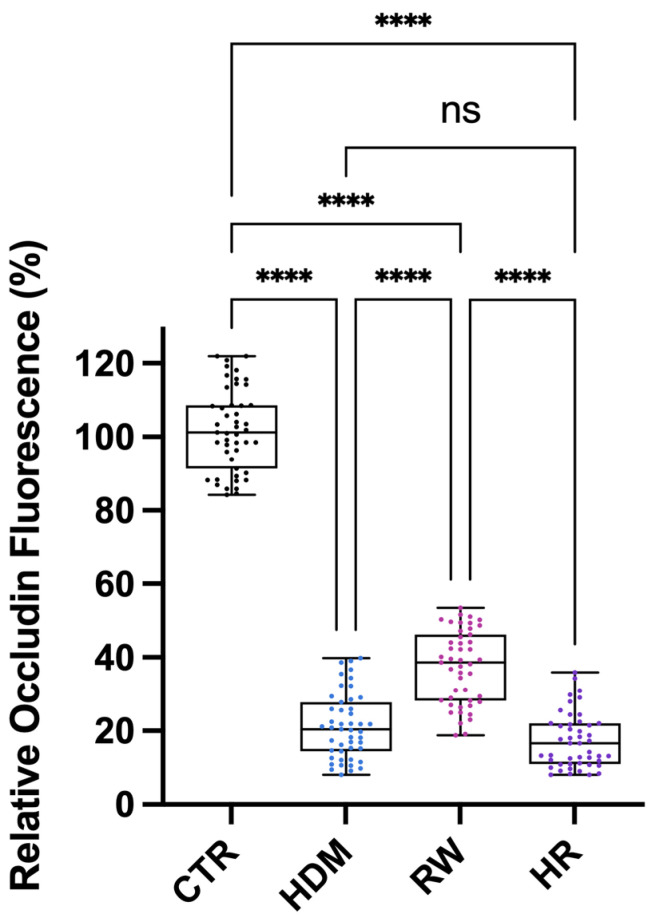
Quantitative analysis of occludin immunofluorescence intensity across treatment groups. Occludin fluorescence intensity was measured from immunofluorescence images of differentiated NHBE cells following allergen exposure, using ImageJ software (version 1.53m). Data are presented as box-and-whisker plots showing median, interquartile range and individual data points, derived from three independent experiments. For each experiment, three representative images per condition were analyzed, with multiple non-overlapping microscopic fields quantified per image. Statistical analysis was performed using one-way ANOVA followed by Tukey’s multiple comparisons test. Significance levels are indicated as **** *p* < 0.0001; ns, not significant. Abbreviations: CTR, control; RW, ragweed pollen extract (100 µg/mL); HDM, house dust mite extract (100 µg/mL); HR, combined exposure of 50 µg/mL HDM + 50 µg/mL RW (total 100 µg/mL).

Quantitative analysis of occludin fluorescence intensity was performed using ImageJ.JS 1.53m software. All allergen-exposed groups (HDM100, RW100 and HR100) exhibited a marked reduction in occludin fluorescence intensity compared with control cells (*p* < 0.0001), indicating significant disruption of tight junction integrity. Among exposed conditions, combined exposure (HR100) demonstrated a more pronounced reduction compared with RW100 (*p* < 0.0001), while no significant difference was observed between HR100 and HDM100. Additional pairwise comparisons between individual allergen exposures revealed significant differences, as illustrated in Figure 6.

### 3.3. Transcriptional Responses Associated with Barrier Disruption and Immune Signaling

**Figure 7:**
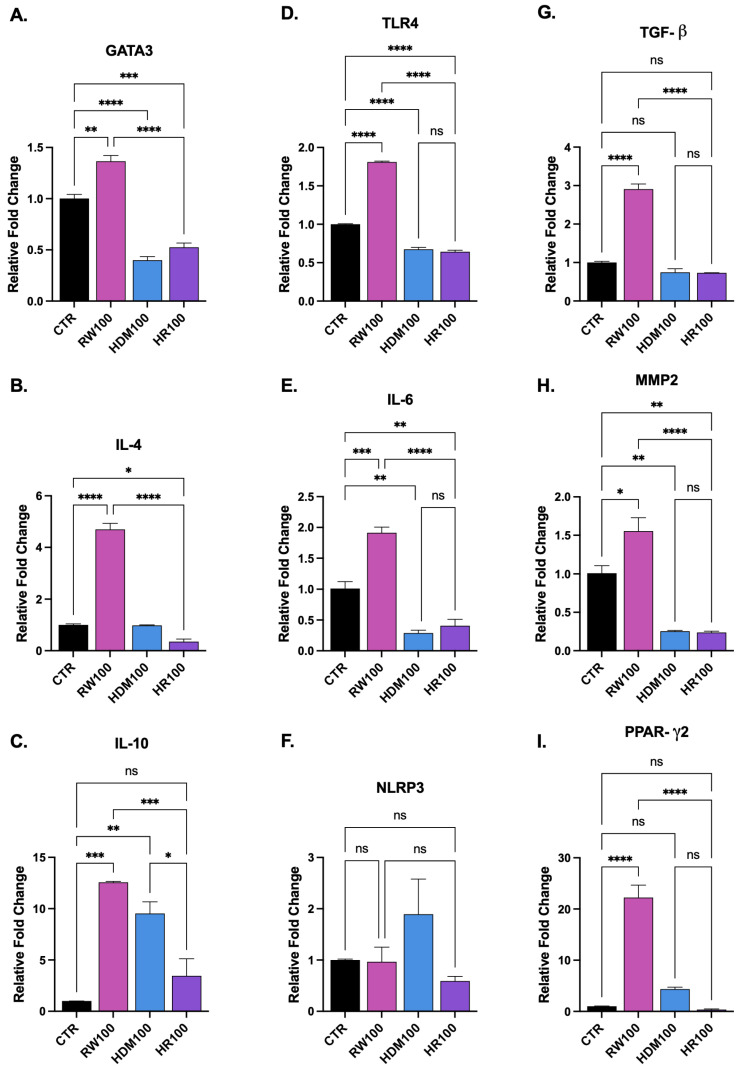
Allergen-induced modulation of immune-, inflammatory- and remodeling-related gene expression in bronchial epithelial cells. Relative mRNA expression levels were quantified by RT-qPCR following allergen exposure and are presented as fold change relative to control (CTR) using the 2^−ΔΔCt^ method after normalization to a housekeeping gene. (**A**–**C**) Genes associated with type 2-related inflammatory responses: GATA3 (**A**), IL-4 (**B**) and IL-10 (**C**). (**D**–**F**) Genes related to innate inflammatory and inflammasome responses: TLR4 (**D**), IL-6 (**E**) and NLRP3 (**F**). (**G**–**I**) Genes associated with epithelial remodeling and metabolic regulation: TGF-β (**G**), MMP2 (**H**) and PPAR-γ2 (**I**). Data are presented as mean ± SEM from three independent experiments performed in duplicate. Statistical comparisons were performed using one-way ANOVA followed by Tukey’s multiple comparisons test. Statistical analyses were conducted on ΔCt values, whereas graphical data are displayed as 2^−ΔΔCt^ fold-change values. Statistical significance is indicated as * *p* < 0.05; ** *p* < 0.01; *** *p* < 0.001; **** *p* < 0.0001; ns, not significant. The groups are labeled as follows: CTR, control; RW100, ragweed pollen extract (100 µg/mL); HDM100, house dust mite extract (100 µg/mL); HR100, combined exposure of 50 µg/mL HDM + 50 µg/mL RW (total 100 µg/mL).

RT-qPCR analysis demonstrated allergen-dependent modulation of genes involved in epithelial barrier regulation, innate immune signaling and airway remodeling (Figure 7). Whereas RW challenge induced coordinated upregulation of genes associated with immune responses, tissue remodeling and metabolic regulation, exposure to HDM and particularly HR was characterized by attenuated or suppressed expression of several analyzed genes, concordant with a stress-associated epithelial phenotype.

#### 3.3.1. Modulation of Genes Associated with Type 2 Inflammatory Responses (Figure 7A–C)

Quantitative PCR was performed to assess the impact of allergen exposure on the genes associated with type 2-related cytokine expression by measuring the relative mRNA levels of IL-4, GATA3 and IL-10. Relative gene expression was calculated using the 2^−ΔΔCt^ method, with GAPDH as endogenous control and normalization to control samples (Figure 2).

RW100 exposure induced robust upregulation of IL-4 and GATA3, confirming upregulation of classical genes associated with type 2-related cytokine expression. IL-4 expression was markedly increased compared with control (*p* < 0.0001) and GATA3 expression was significantly elevated (*p* = 0.0017). In contrast, HDM100 and HR100 failed to induce comparable Th2 activation. No significant difference in IL-4 expression was observed with HDM100 (*p* = 0.9996) and was only modestly but significant increased under HR100 (*p* = 0.0302), with both conditions showing significantly lower expression compared with RW100 (*p* < 0.0001). GATA3 expression was significantly reduced under both HDM100 and HR100 relative to control (*p* < 0.0001 and *p* = 0.0003, respectively), suggesting impaired transcriptional polarization.

A similar suppressive trend was observed for the regulatory cytokine IL-10. Although RW100 and HDM100 significantly increased IL-10 expression (*p* = 0.0002 and *p* = 0.0014), HR100 failed to induce IL-10 and did not differ from control (*p* = 0.3683). Both RW100 and HDM100 exhibited significantly higher IL-10 levels than HR100 (*p* = 0.0009 and *p* = 0.0115, respectively). Together, these findings indicate that while RW triggers an active type 2-associated transcriptional profile, HDM and especially HR exposure blunt both effector and regulatory cytokine responses, suggesting epithelial functional impairment under combined allergen challenge.

#### 3.3.2. Gene Expression Related to Inflammatory and Inflammasome Responses (Figure 7D–F)

To assess epithelial innate immune activation following allergen exposure, the relative mRNA expression levels of TLR4, IL-6 and NLRP3 were quantified.

Expression of TLR4, a key epithelial pattern recognition receptor, was significantly increased following RW100 compared with control (*p* < 0.0001). In contrast, both HDM100 and HR100 exhibited significantly lower TLR4 expression than control (both *p* < 0.0001) and were markedly lower than RW100 (*p* < 0.0001).

A similar pattern was observed for IL-6, with RW100 significantly increasing IL-6 expression relative to control (*p* = 0.0005), whereas HDM100 and HR100 exhibited significantly reduced expression relative to control conditions (*p* = 0.0023 and *p* = 0.0070, respectively). No significant difference was observed between HDM100 and HR100 (*p* = 0.7939). RW100 expression levels were significantly higher than both HDM100 and HR100 (both *p* < 0.0001).

Expression of the inflammasome-associated gene NLRP3 did not differ significantly among experimental groups (*p* > 0.05). These findings suggest that inflammasome activation may not represent a dominant epithelial response under the exposure conditions tested, although inflammasome signaling may become more prominent in the presence of immune cell interactions, which were not included in the monoculture epithelial model used in this study.

#### 3.3.3. Modulation of Epithelial Remodeling and Metabolic Regulatory Responses Following Allergen Exposure (Figure 7G–I)

To further characterize epithelial responses to allergen exposure, the relative mRNA expression levels of TGF-β, PPAR-γ2 and MMP2 were quantified.

Following RW pollen extract exposure, the gene expression was significantly increased for TGF-β (*p* < 0.0001), PPAR-γ2 (*p* < 0.0001) and MMP2 (*p* = 0.0206) compared with control, indicating activation of tissue remodeling and metabolic regulatory programs. In contrast, HDM100 and HR100 failed to significantly induce TGF-β or PPAR-γ2 and did not differ from control (*p* > 0.05), with expression levels significantly lower than RW100 (*p* < 0.0001). MMP2 expression was significantly reduced under HDM100 and HR100 compared with control (*p* = 0.0031 and *p* = 0.0027, respectively), with no difference between these two conditions.

This broad attenuation of remodeling and metabolic signaling under HDM and HR exposure further supports the presence of a compromised epithelial transcriptional state.

These findings should be interpreted as epithelial-derived transcriptional responses within an isolated in vitro system, without direct inference on systemic immune signaling.

## 4. Discussion

This study provides an integrated evaluation of allergen-induced airway epithelial barrier dysfunction by combining functional (TEER and xCELLigence assessment), structural (tight junction imaging) and molecular analyses in differentiated NHBE cell cultures. Unlike previous studies focusing on single readouts or individual allergens, our findings demonstrate that cumulative allergen exposure produces amplified and sustained barrier disruption across multiple biological levels. These results support emerging concepts positioning epithelial barrier dysfunction as a central driver of allergic airway disease rather than a secondary consequence [12,38,39,40].

### 4.1. Functional Implications of Cumulative Allergen Exposure on Epithelial Barrier Integrity

TEER represents a validated functional measure of paracellular permeability in bronchial epithelial models and reflects the coordinated contribution of tight junction complexes to epithelial cohesion [35,41,42].

In the context of NHBE exposure to HDM and RW extracts, TEER serves as an integrative functional readout that captures the cumulative impact of allergen-driven signaling on epithelial barrier integrity. Sustained reductions in TEER reflect compromised barrier integrity that may facilitate allergen penetration, promote epithelial immune activation and amplification of airway inflammation [35,43,44].

AUC analysis confirms and extends the time-course findings by demonstrating that allergen exposure induces not only transient alterations but also significant cumulative impairment in epithelial function. The progressive decline in AUC values across increasing doses and combined exposures provides integrated quantitative evidence for dose-dependent and interaction-driven barrier dysfunction, supporting the concept that cumulative allergen burden critically shapes epithelial resilience over time [18,45]. Importantly, the magnitude of TEER reduction observed under combined allergen exposure exceeded that induced by individual allergens, indicating a greater-than-additive effect on epithelial barrier disruption. This suggests that simultaneous exposure to multiple aeroallergens may overwhelm epithelial adaptive responses and accelerate junctional destabilization. The cumulative reduction in barrier function captured by AUC analysis may reflect persistent epithelial vulnerability, a feature associated with enhanced allergen penetration and chronic inflammatory activation in allergic airway disease.

The functional data derived from TEER and xCELLigence consistently demonstrate that combined exposure to RW and HDM allergens induces more severe and sustained barrier dysfunction than exposure to individual allergens as discussed in previous studies [18,35]. The initial transient increase in TEER observed across conditions likely reflects an early adaptive response involving cytoskeletal reorganization and transient tightening of tight junction complexes, processes previously described following epithelial stress exposure [46].

However, this adaptive phase was followed by a pronounced and persistent decline in barrier integrity, particularly under combined allergen exposure. These findings suggest that cumulative allergen challenge exceeds the adaptive capacity of the epithelium, leading to progressive junctional impairment and loss of epithelial cohesion. The concordance between TEER and xCELLigence readouts strengthens the interpretation that these changes reflect genuine functional barrier impairment rather than isolated alterations in cell morphology or adhesion. Also, the absence of a marked decline in cell index suggests that the observed barrier alterations occur in the absence of overt cytotoxicity.

### 4.2. Tight Junction Alterations as a Structural Basis for Barrier Dysfunction

Structural analysis by occludin immunofluorescence revealed marked alterations of tight junction architecture following allergen exposure. The progressive fragmentation and discontinuity of occludin localization observed under combined allergen conditions provide a clear structural correlation for the functional barrier impairment detected by TEER and impedance measurements [47]. The marked occludin redistribution observed in HR-treated cultures is consistent with the pronounced TEER decline detected in parallel, supporting a direct relationship between junctional disorganization and functional barrier impairment. These alterations consistent with protease-mediated cleavage, junctional protein redistribution and cytoskeletal destabilization described for both pollen- and mite-derived allergens [16,48,49,50].

Notably, single-allergen exposure resulted in more localized and less extensive junctional disruption, suggesting partial preservation of epithelial architecture compared to the allergen co-exposure. This differential structural response supports the concept that allergen co-exposure produces greater-than-additive effects on epithelial junctional stability [18,32,51,52]. A similar interaction pattern was observed for both TEER- and impedance-derived measurements, supporting the robustness of the co-exposure effect across complementary functional readouts. Moreover, the significant RW × HDM interaction and the greater magnitude of barrier impairment under HR conditions were consistent with a greater-than-additive response, suggestive of a synergistic-like interaction. Such effect is highly relevant in real-world exposure scenarios, where individuals are rarely exposed to a single allergen [53,54].

### 4.3. Transcriptional Reprogramming of the Epithelium Under Combined Allergen Stress

At the molecular level, RT-qPCR analysis demonstrated allergen-dependent transcriptional modulation of genes involved in epithelial immune signaling, barrier regulation and tissue remodeling, with gene expression profiles varying according to the type of stimulus rather than exhibiting uniform changes. RW pollen extract exposure elicited coordinated upregulation of multiple axes, suggesting an active and responsive epithelial state. In contrast, HDM and especially combined HR exposure were characterized by broad attenuation of gene expression across multiple signaling axes, suggesting transcriptional suppression rather than activation. This pattern aligns with the functional and structural data and supports the concept of epithelial stress-induced dysfunction under cumulative allergen burden, reflecting adaptive responses to barrier perturbation and immune activation [55,56,57].

Allergen exposure markedly influenced the transcription of mediators involved in epithelial inflammatory signaling. The increased expression of IL-6 following ragweed exposure suggests enhanced pro-inflammatory responses associated with leukocyte recruitment and amplification of local immune activity, whereas the upregulation of TLR4 indicates increased epithelial responsiveness to environmental stimuli and allergen-associated molecular patterns [39,58,59,60]. In contrast, both HDM and HR exposure resulted in reduced TLR4 and IL-6 expression compared to control. Rather than merely reflecting differential allergen recognition, this attenuation may indicate impaired transcriptional responsiveness associated with epithelial stress. Given that HDM and HR exposure were also associated with greater barrier dysfunction in TEER and impedance analyses, the reduced innate signaling likely reflects a state of functional compromise rather than diminished challenge.

RW exposure robustly increased IL-4 and GATA3 expression, indicating modulation of genes associated with type 2 immune responses. RW also induced IL-10 expression, indicating engagement of compensatory regulatory mechanisms that may temper excessive inflammation. In contrast, HDM and HR groups displayed a markedly attenuated transcriptional profile. GATA3 expression was significantly reduced, and IL-4 induction was minimal or modest compared with ragweed. Most strikingly, HR exposure failed to induce IL-10, suggesting a loss of regulatory capacity under combined allergen stress [59,61,62]. The simultaneous reduction in both effector (IL-4, GATA3) and regulatory (IL-10) mediators under HR conditions suggests an overall decrease in gene expression rather than selective effects on specific immune mediators. This pattern supports the hypothesis that cumulative allergen exposure imposes a greater stress-associated epithelial dysfunction, limiting their capacity to mount coordinated immune responses.

Genes associated with epithelial injury and tissue remodeling were also differentially regulated. Although NLRP3 expression did not differ significantly among experimental groups, indicating minimal modulation of inflammasome-related gene expression at the evaluated time point, significant changes were observed in genes associated with structural remodeling and tissue repair [63]. Increased expression of TGF-β and MMP2 following ragweed exposure suggests enhanced expression of genes associated with extracellular matrix turnover, epithelial repair and airway structural remodeling [64,65]. These molecular alterations suggest epithelial responses to injury and may contribute to long-term structural changes in the airway epithelium. In contrast, HDM and HR exposure failed to induce TGF-β and PPAR-γ2 and were associated with reduced MMP2 expression. The absence of remodeling and metabolic compensation under HR conditions further supports the presence of epithelial dysfunction rather than adaptive repair.

Furthermore, increased PPAR-γ2 expression suggests a potential role for immunometabolic regulation in epithelial responses to allergen exposure. Given the known involvement of PPARγ in anti-inflammatory responses, lipid metabolism and epithelial homeostasis, its reduced expression under combined allergen exposure may contribute to sustained epithelial instability and impaired recovery [66,67]. The molecular attenuation observed in HDM and HR groups, therefore, parallels the more pronounced barrier disruption detected functionally (TEER, impedance) and structurally (occludin redistribution) [68].

Beyond transcriptional changes, post-transcriptional regulation by microRNAs represents an additional layer controlling epithelial barrier integrity and immune responses in asthma. Specific miRNAs, including miR-21, miR-155 and the let-7 family, modulate cytokine signaling, T-helper cell polarization and tight junction protein expression, while also influencing epithelial-mesenchymal transition and tissue remodeling processes [69]. Through these mechanisms, miRNAs may contribute to sustained epithelial dysfunction and represent potential biomarkers or therapeutic targets in allergic airway disease.

The convergence of transcriptional alterations with functional and structural findings emphasizes the multi-layered nature of epithelial barrier dysfunction. The transcriptional attenuation observed under HDM and HR conditions paralleled the functional impairment demonstrated by TEER decline and reduced impedance-based cell index, as well as with tight junction remodeling observed by occludin immunofluorescence. Together, these findings suggest that epithelial barrier dysfunction under cumulative allergen exposure is not merely structural but involves a coordinated collapse of transcriptional programs governing immune recognition, regulatory signaling and tissue repair, reinforcing epithelial dysfunction and sustaining inflammatory responses over time [70,71].

### 4.4. Pathophysiological and Translational Implications

From a pathophysiological perspective, these findings support a model in which cumulative allergen exposure progressively destabilizes the airway epithelial barrier, thereby facilitating enhanced allergen penetration and amplifying epithelial-immune crosstalk. Such barrier compromise may lower the threshold for immune activation, contribute to chronic airway inflammation and promote disease persistence or severity in allergic asthma [43,72,73].

Beyond local epithelial responses, systemic factors may further modulate barrier integrity and inflammatory signaling. Recent findings increasingly highlight the role of microbiota-mediated immune regulation in asthma pathogenesis, emphasizing the importance of the gut-lung axis in controlling airway inflammation and epithelial barrier function [4,74,75]. Alterations in microbial composition and microbe-derived metabolites can influence cytokine signaling, epithelial activity and systemic inflammatory responses, thereby contributing to disease heterogeneity and variability in therapeutic outcomes [76].

Importantly, the multimodal approach employed in this study provides a framework for future investigations aimed at analyzing epithelial-driven mechanisms in allergic airway disease. By capturing multilateral dimensions of barrier dysfunction simultaneously, this strategy may prove valuable for evaluating therapeutic interventions targeting epithelial resilience and barrier restoration [77,78].

A key finding of this study is that combined allergen exposure did not amplify epithelial inflammatory responses but instead resulted in a broad reduction in the expression of genes associated with immune regulation and tissue remodeling. Rather than reflecting enhanced inflammatory amplification, co-exposure to RW and HDM was associated with reduced mRNA expression of genes involved in innate sensing (TLR4, IL-6), type 2-related gene expression (GATA3, IL-4), regulatory cytokine responses (IL-10) and remodeling or metabolic regulation (TGF-β, PPARγ2, MMP2). When interpreted alongside the pronounced barrier dysfunction observed in TEER, impedance analysis and occludin redistribution, this pattern suggests epithelial destabilization under cumulative allergen burden.

In real-world settings, simultaneous exposure to multiple aeroallergens is common. The present data indicate that cumulative allergen stress may impose a greater biological burden than individual stimuli, leading to impaired transcriptional competence and reduced adaptive capacity [18,69,78]. Suppression of both effector (IL-4, GATA3) and regulatory (IL-10, PPARγ2) mediators under combined exposure suggests loss of epithelial immune balance and diminished capacity for barrier restoration. The pronounced impairment observed following combined allergen exposure emphasizes the importance of studying barrier biology in the context of multi-allergen environments, which may produce cumulative stress that amplifies barrier dysfunction beyond the effects of individual allergens.

The present findings may also be relevant within the broader context of allergic disease endotyping and precision medicine approaches. Although allergic respiratory diseases are traditionally interpreted predominantly through IgE-mediated mechanisms, increasing evidence suggests that epithelial dysfunction, innate immune activation and non-IgE-associated inflammatory pathways contribute substantially to disease heterogeneity and clinical expression [1,71,79]. Airway epithelial cells actively participate in immune regulation through the release of alarmins, cytokines and stress-associated mediators capable of amplifying inflammatory responses independently of classical allergen-specific IgE signaling [80]. In this regard, the pronounced epithelial alterations observed following combined allergen exposure in our model may reflect biological mechanisms involved in complex allergen-driven inflammatory responses that extend beyond isolated adaptive IgE-mediated pathways. Experimental approaches such as the Leukocyte Adherence Inhibition Test (LAIT) and Tube Titration of Precipitins (TTP) have been proposed as complementary tools for investigating non-IgE-mediated immunoreactivity and individualized immune response patterns in allergic diseases [19,21].

The enhanced barrier impairment induced by combined HDM and RW exposure in our study may be particularly relevant in patients with persistent respiratory symptoms that are insufficiently explained by classical IgE-mediated sensitization patterns, supporting the need for broader integrative models of allergic disease pathophysiology.

From a translational perspective, these findings reinforce the concept that epithelial dysfunction in allergic airway disease is not solely driven by inflammatory amplification but may also reflect stress-associated functional impairment. Targeting epithelial resilience and barrier restoration mechanisms may therefore represent rational therapeutic strategies, particularly in multi-sensitized patients.

Together, these findings support a model in which cumulative allergen exposure drives epithelial dysfunction through functional impairment and transcriptional attenuation rather than classical inflammatory amplification, highlighting epithelial resilience as a central determinant of disease progression.

### 4.5. Implications of Airway Epithelial Barrier Dysfunction for Systemic and Vascular Inflammation

Although the present study focuses on airway epithelial pathology, epithelial barrier dysfunction may have consequences beyond the local airway environment. Disruption of epithelial integrity following allergen exposure facilitates increased permeability and may promote inflammatory responses, potentially allowing epithelial-derived mediators to enter systemic circulation [81].

Airway epithelial barrier dysfunction represents an important early event in the pathophysiology of allergic asthma, with implications that extend beyond localized airway injury [3,7]. Endothelial activation involves NF-κB-dependent upregulation of adhesion molecules and pro-inflammatory cytokines, leading to enhanced vascular permeability and immune cell recruitment. In this context, chronic epithelial barrier impairment in allergic airway disease may contribute to systemic inflammatory tone and vascular dysregulation [82,83].

While direct vascular outcomes were not evaluated in this study, the convergence of functional barrier decline, tight junction disorganization and transcriptional modulation supports a mechanistic basis for future investigations exploring the lung-vascular axis in allergic disease.

### 4.6. Study Limitations and Future Directions

While this study offers detailed insight into allergen-induced epithelial barrier dysfunction, certain limitations should be acknowledged. First, the in vitro design enables controlled investigation of epithelial-specific responses but does not fully recapitulate the complexity of the in vivo airway microenvironment, including immune cell interactions, vascular components and systemic regulatory mechanisms. As a result, epithelial-immune crosstalk and downstream systemic consequences could not be directly assessed.

Second, molecular analyses were primarily limited to gene expression and did not include additional quantitative protein-level validation beyond occludin immunofluorescence, which restricts confirmation of downstream signaling activity and effector responses. Although occludin immunofluorescence provided structural protein-level support for tight junction disruption, complementary quantitative approaches such as Western blotting, multiplex cytokine assays or proteomic analyses would further strengthen the mechanistic interpretation of the transcriptional findings. Third, the experimental framework focused on short-term allergen exposure and therefore may not fully capture the chronic inflammatory processes and structural remodeling characteristic of persistent allergic airway disease.

The selection of 100 µg/mL and 72 h as the single RT-qPCR condition was guided by functional data demonstrating maximal barrier disruption at this concentration and time point, enabling direct integration of transcriptional findings with the observed functional and structural alterations. However, dose-response transcriptional profiling, particularly at lower concentrations (e.g., 10 µg/mL), could reveal threshold-dependent signaling events and early mechanistic responses not captured at the concentration associated with maximal barrier impairment. Similarly, earlier time points (e.g., 6–24 h) may detect transient cytokine signaling, particularly for rapidly induced genes such as IL-6 and TLR4, which may peak early. Several of the analyzed genes, including TGF-β, MMP2, PPAR-γ2 and GATA3, are primarily associated with sustained tissue remodeling and epithelial stress responses, supporting the relevance of the 72-h time point for their evaluation.

In vivo validation using established models of allergic airway disease would provide translational relevance by integrating epithelial responses within a complete immune and systemic context. Longitudinal experimental designs incorporating multiple time points and allergen concentrations would further clarify the temporal and dose-dependent dynamics of epithelial signaling.

Although the present study did not directly investigate allergen-specific immune endotypes, the multimodal framework employed here supports the concept that epithelial barrier dysfunction represents an important upstream component of allergic disease heterogeneity. The integration of functional barrier assessment, structural junctional analysis and transcriptional profiling may contribute to a more comprehensive characterization of epithelial response patterns associated with different environmental exposures. Such approaches may ultimately support future precision medicine strategies aimed at identifying biologically distinct airway disease phenotypes and developing mechanism-based therapeutic interventions tailored to specific inflammatory and epithelial dysfunction profiles.

Moreover, the regulatory mechanisms underlying epithelial responses to allergen exposure are likely more complex than captured by targeted analyses. Transcriptomic analyses, including bulk or single-cell RNA sequencing, would enable identification of novel regulatory networks and non-coding RNA species involved in epithelial stress responses following allergen exposure. Complementary proteomic or secretome analyses could capture post-transcriptional events not reflected in mRNA expression, including changes in protein turnover, alarmin release and extracellular matrix composition. Integrative multi-omics approaches, combining transcriptomic, proteomic and metabolomic data, have shown promise in characterizing epithelial barrier responses in related contexts and would provide a systems-level understanding of the regulatory architecture underlying allergen-induced barrier dysfunction, as well as the identification of additional pathways and candidate biomarkers associated with barrier disruption [78].

The incorporation of co-culture systems in future studies would represent a valuable extension of the present experimental framework, enabling the investigation of epithelial–immune interactions and early alarmin signaling events, including IL-33, TSLP and IL-25. This would provide a more comprehensive understanding of early immune activation and epithelial–immune communication following allergen exposure. These limitations define several important directions for future research. Nevertheless, the controlled multimodal experimental design enabled precise characterization of epithelial-specific mechanisms that are difficult to isolate in complex in vivo systems.

## 5. Conclusions

This study provides a multimodal framework for characterizing allergen-induced airway epithelial barrier dysfunction through integrated functional, structural and molecular analyses. Across complementary readouts, including TEER, real-time impedance monitoring, occludin immunofluorescence, and transcriptional profiling, combined exposure to RW pollen and HDM extracts induced the most pronounced and sustained impairment of epithelial integrity, as demonstrated by both temporal dynamics and cumulative (AUC) responses. This was accompanied by disruption of TJ organization and broad alterations in epithelial gene expression across immune, regulatory and remodeling pathways. Notably, combined allergen exposure resulted in barrier impairment exceeding that induced by individual allergens, with important implications for real-world environmental exposures. These findings point to mechanisms that extend beyond simple additive inflammatory effects, reflecting reduced epithelial resilience and promoting persistent allergen penetration. Beyond demonstrating the detrimental effects of combined allergen exposure on airway epithelial integrity, these findings also support the emerging concept that epithelial dysfunction and innate inflammatory signaling contribute to allergic disease heterogeneity beyond classical IgE-mediated mechanisms. Taken together, the results support the concept of the airway epithelium as an active regulator of allergic airway disease, in which barrier dysfunction represents an early pathogenic event, and highlight that cumulative allergen exposure imposes a disproportionate biological burden. The multimodal framework presented here provides a valuable platform for future investigations and supports epithelial barrier restoration as a promising therapeutic strategy.

## Figures and Tables

**Table 1:** RT-qPCR primer sequences.

Gene	Forward Primer	Reverse Primer
IL-4	CCGTAACAGACATCTTTGCTGCC	GAGTGTCCTTCTCATGGTGGCT
IL-6	AGACAGCCACTCACCTCTTCAG	TTCTGCCAGTGCCTCTTTGCTG
IL-10	TCTCCGAGATGCCTTCAGCAGA	TCAGACAAGGCTTGGCAACCCA
TLR4	CCCTGAGGCATTTAGGCAGCTA	AGGTAGAGAGGTGGCTTAGGCT
GATA3	ACCACAACCACACTCTGGAGGA	TCGGTTTCTGGTCTGGATGCCT
TGF-β	GACTGCGGATCTCTGTGTCA	CCTCCCTTAACCTCTCTGGG
MMP2	ACCTGGATGCCGTCGTGGAC	GTGGCAGCACCAGGGCAGC
NLRP3	GGACTGAAGCACCTGTTGTGCA	TCCTGAGTCTCCCAAGGCATTC
PPAR-γ2	GAATGTCGTGTCTGTGGAGA	TGAGGAGAGTTACTTGGTCG
GAPDH	GTCTCCTCTGACTTCAACAGCG	ACCACCCTGTTGCTGTAGCCAA

## Data Availability

The original contributions presented in this study are included in the article; further inquiries can be directed to the corresponding author.

## References

[1] Hewitt R.J., Lloyd C.M. (2021). Regulation of Immune Responses by the Airway Epithelial Cell Landscape. Nat. Rev. Immunol..

[2] Varricchi G., Brightling C.E., Grainge C., Lambrecht B.N., Chanez P. (2024). Airway Remodelling in Asthma and the Epithelium: On the Edge of a New Era. Eur. Respir. J..

[3] Meng J., Xiao H., Xu F., She X., Liu C., Canonica G.W. (2025). Systemic Barrier Dysfunction in Type 2 Inflammation Diseases: Perspective in the Skin, Airways, and Gastrointestinal Tract. Immunol. Res..

[4] Pat Y., Yazici D., D’Avino P., Li M., Ardicli S., Ardicli O., Mitamura Y., Akdis M., Dhir R., Nadeau K. (2024). Recent Advances in the Epithelial Barrier Theory. Int. Immunol..

[5] Frey A., Lunding L.P., Ehlers J.C., Weckmann M., Zissler U.M., Wegmann M. (2020). More Than Just a Barrier: The Immune Functions of the Airway Epithelium in Asthma Pathogenesis. Front. Immunol..

[6] Russell R.J., Boulet L.-P., Brightling C.E., Pavord I.D., Porsbjerg C., Dorscheid D., Sverrild A. (2024). The Airway Epithelium: An Orchestrator of Inflammation, a Key Structural Barrier and a Therapeutic Target in Severe Asthma. Eur. Respir. J..

[7] Georas S.N., Rezaee F. (2014). Epithelial Barrier Function: At the Front Line of Asthma Immunology and Allergic Airway Inflammation. J. Allergy Clin. Immunol..

[8] Holgate S.T. (2012). Innate and Adaptive Immune Responses in Asthma. Nat. Med..

[9] Soh W.T., Zhang J., Hollenberg M.D., Vliagoftis H., Rothenberg M.E., Sokol C.L., Robinson C., Jacquet A. (2023). Protease Allergens as Initiators–Regulators of Allergic Inflammation. Allergy.

[10] Jacquet A. (2011). The Role of Innate Immunity Activation in House Dust Mite Allergy. Trends Mol. Med..

[11] Wimmer M., Alessandrini F., Gilles S., Frank U., Oeder S., Hauser M., Ring J., Ferreira F., Ernst D., Winkler J.B. (2015). Pollen-derived Adenosine Is a Necessary Cofactor for Ragweed Allergy. Allergy.

[12] Akdis C.A. (2021). Does the Epithelial Barrier Hypothesis Explain the Increase in Allergy, Autoimmunity and Other Chronic Conditions?. Nat. Rev. Immunol..

[13] Jacquet A. (2021). Characterization of Innate Immune Responses to House Dust Mite Allergens: Pitfalls and Limitations. Front. Allergy.

[14] Bergmann K.-C. (2022). Frequency of Sensitizations and Allergies to House Dust Mites. Allergo J. Int..

[15] Grijincu M., Huțu I., Weber M., Babaev E., Stolz F., Valenta R., Păunescu V., Panaitescu C., Chen K.-W. (2023). Physicochemical and Immunological Characterization of Amb a 12, a Novel Ragweed (*Ambrosia artemisiifolia*) Pollen Allergen. Mol. Immunol..

[16] Ogi K., Ramezanpour M., Liu S., Ferdoush Tuli J., Bennett C., Suzuki M., Fujieda S., Psaltis A.J., Wormald P.-J., Vreugde S. (2021). Der p 1 Disrupts the Epithelial Barrier and Induces IL-6 Production in Patients With House Dust Mite Allergic Rhinitis. Front. Allergy.

[17] Huang Z., Liu J., Sun L., Ong H.H., Ye J., Xu Y., Wang D. (2024). Updated Epithelial Barrier Dysfunction in Chronic Rhinosinusitis: Targeting Pathophysiology and Treatment Response of Tight Junctions. Allergy.

[18] Zimbru R.-I., Grijincu M., Tănasie G., Zimbru E.-L., Bojin F.-M., Buzan R.-M., Tamaș T.-P., Cotarcă M.-D., Harich O.O., Pătrașcu R. (2025). Breaking Barriers: The Detrimental Effects of Combined Ragweed and House Dust Mite Allergen Extract Exposure on the Bronchial Epithelium. Appl. Sci..

[19] Olivier C.E., Pinto D.G., Teixeira A.P.M., Miguel C.S., Santos R.A.P.G., Rocha N.S.D., Santana J.L.S., Lima R.P.S. (2025). Endotyping Cellular and Humoral Immunoreactivity against Pollen and Citrus Fruits in Patients with Non–IgE-Mediated Rhinoconjunctivitis. Asian J. Immunol..

[20] Ogulur I., Mitamura Y., Yazici D., Pat Y., Ardicli S., Li M., D’Avino P., Beha C., Babayev H., Zhao B. (2025). Type 2 Immunity in Allergic Diseases. Cell. Mol. Immunol..

[21] Olivier C.E., Pinto D.G., Teixeira A.P.M., Santana J.L.S., Santos R.A.P.G., Lima R.P.S., Monteiro E.S. (2024). Endotyping Non IgE Mediated Immunoreactivity to *Dermatophagoides farinae*: Implications for Allergic Patients: A Retrospective Study. Asian J. Immunol..

[22] Castillo E.F., Zheng H., Yang X.O. (2018). Orchestration of Epithelial-Derived Cytokines and Innate Immune Cells in Allergic Airway Inflammation. Cytokine Growth Factor Rev..

[23] Losol P., Ji M.-H., Kim J.H., Choi J.-P., Yun J.-E., Seo J.-H., Kim B.-K., Chang Y.-S., Kim S.-H. (2023). Bronchial Epithelial Cells Release Inflammatory Markers Linked to Airway Inflammation and Remodeling in Response to TLR5 Ligand Flagellin. World Allergy Organ. J..

[24] De Lagarde V.M., Chevalier L., Méausoone C., Cazier F., Dewaele D., Cazier-Dennin F., Janona M., Logie C., Achard S., André V. (2024). Acute and Repeated Exposures of Normal Human Bronchial Epithelial (NHBE) Cells Culture to Particles from a Coloured Pyrotechnic Smoke. Environ. Toxicol. Pharmacol..

[25] Mari M., Monteleone G., Conte M., Confalonieri P., Salton F., Antonaglia C., Galantino A., Reccardini N., Hughes M., Geri P. (2026). Effectiveness of Desensitization Therapy for Grasses and Dust Mites on Asthma Exacerbations and Respiratory Function During the Allergy Season: A Pilot Study. J. Clin. Med..

[26] Hesse L., Petersen A.H., Nawijn M.C. (2021). Methods for Experimental Allergen Immunotherapy: Subcutaneous and Sublingual Desensitization in Mouse Models of Allergic Asthma. Methods Mol. Biol..

[27] Zhou J., Zhou X., Xu R., Du X., Li Q., Li B., Zhang G., Chen L., Perelman J.M., Kolosov V.P. (2021). The Degradation of Airway Epithelial Tight Junctions in Asthma Under High Airway Pressure Is Probably Mediated by Piezo-1. Front. Physiol..

[28] Kim B.G., Lee P.H., Lee S.H., Baek A.R., Park J.S., Lee J., Park S.W., Kim D.J., Park C.S., Jang A.S. (2018). Impact of the Endothelial Tight Junction Protein Claudin-5 on Clinical Profiles of Patients With COPD. Allergy Asthma Immunol. Res..

[29] Nazari H., Shrestha J., Naei V.Y., Bazaz S.R., Sabbagh M., Thiery J.P., Warkiani M.E. (2023). Advances in TEER Measurements of Biological Barriers in Microphysiological Systems. Biosens. Bioelectron..

[30] Haidar L., Bănărescu C.F., Uța C., Zimbru E.-L., Zimbru R.-I., Tîrziu A., Pătrașcu R., Șerb A.-F., Georgescu M., Nistor D. (2025). Beyond the Skin: Exploring the Gut–Skin Axis in Chronic Spontaneous Urticaria and Other Inflammatory Skin Diseases. Biomedicines.

[31] Alexandru I., Nistor D., Motofelea A.C., Cadar B.-A., Crintea A., Tatu C., Pop G.N., Csep A.N. (2024). Vitamins, Coenzyme Q10, and Antioxidant Strategies to Improve Oocyte Quality in Women with Gynecological Cancers: A Comprehensive Review. Antioxidants.

[32] Zimbru R.-I., Zimbru E.-L., Ordodi V.-L., Bojin F.-M., Crîsnic D., Grijincu M., Mirica S.-N., Tănasie G., Georgescu M., Huțu I. (2024). The Impact of High-Fructose Diet and Co-Sensitization to House Dust Mites and Ragweed Pollen on the Modulation of Airway Reactivity and Serum Biomarkers in Rats. Int. J. Mol. Sci..

[33] Buzan M.-R., Grijincu M., Zbîrcea L.-E., Haidar L., Tamaș T.-P., Cotarcă M.-D., Tănasie G., Weber M., Babaev E., Stolz F. (2024). Insect Cell-Expressed Major Ragweed Allergen Amb a 1.01 Exhibits Similar Allergenic Properties to Its Natural Counterpart from Common Ragweed Pollen. Int. J. Mol. Sci..

[34] Buzan M., Zbîrcea L., Gattinger P., Babaev E., Stolz F., Valenta R., Păunescu V., Panaitescu C., Chen K. (2022). Complex IgE Sensitization Patterns in Ragweed Allergic Patients: Implications for Diagnosis and Specific Immunotherapy. Clin. Transl. Allergy.

[35] Srinivasan B., Kolli A.R., Esch M.B., Abaci H.E., Shuler M.L., Hickman J.J. (2015). TEER Measurement Techniques for In Vitro Barrier Model Systems. SLAS Technol..

[36] Da Conceição Braga L., Gonçalves B.Ô.P., Coelho P.L., Da Silva Filho A.L., Silva L.M. (2022). Identification of Best Housekeeping Genes for the Normalization of RT-qPCR in Human Cell Lines. Acta Histochem..

[37] Eisenberg E., Levanon E.Y. (2013). Human Housekeeping Genes, Revisited. Trends Genet..

[38] Holgate S.T. (2011). The Sentinel Role of the Airway Epithelium in Asthma Pathogenesis. Immunol. Rev..

[39] Fahy J.V. (2015). Type 2 Inflammation in Asthma—Present in Most, Absent in Many. Nat. Rev. Immunol..

[40] Celebi Sözener Z., Cevhertas L., Nadeau K., Akdis M., Akdis C.A. (2020). Environmental Factors in Epithelial Barrier Dysfunction. J. Allergy Clin. Immunol..

[41] Sharma M., Huber E., Arnesdotter E., Behrsing H.P., Bettmann A., Brandwein D., Constant S., Date R., Deshpande A., Fabian E. (2025). Minimum Information for Reporting on the TEER (Trans-Epithelial/Endothelial Electrical Resistance) Assay (MIRTA). Arch. Toxicol..

[42] Lu H., Xiang J., Zhou X., Lin M., Huang C., Yi F., Chen Z., Lai K. (2025). Alteration of the Secretome in Airway Epithelial Cells by Air Pollutants: Evidence from an Air–Liquid Interface Model. Lung.

[43] Hellings P.W., Steelant B. (2020). Epithelial Barriers in Allergy and Asthma. J. Allergy Clin. Immunol..

[44] Davis J.D., Wypych T.P. (2021). Cellular and Functional Heterogeneity of the Airway Epithelium. Mucosal Immunol..

[45] Hamidi H., Lilja J., Ivaska J. (2017). Using xCELLigence RTCA Instrument to Measure Cell Adhesion. Bio-Protocol.

[46] Xiao C., Puddicombe S.M., Field S., Haywood J., Broughton-Head V., Puxeddu I., Haitchi H.M., Vernon-Wilson E., Sammut D., Bedke N. (2011). Defective Epithelial Barrier Function in Asthma. J. Allergy Clin. Immunol..

[47] Noureddine N., Chalubinski M., Wawrzyniak P. (2022). The Role of Defective Epithelial Barriers in Allergic Lung Disease and Asthma Development. J. Asthma Allergy.

[48] Wawrzyniak P., Wawrzyniak M., Wanke K., Sokolowska M., Bendelja K., Rückert B., Globinska A., Jakiela B., Kast J.I., Idzko M. (2017). Regulation of Bronchial Epithelial Barrier Integrity by Type 2 Cytokines and Histone Deacetylases in Asthmatic Patients. J. Allergy Clin. Immunol..

[49] Rauter I., Krauth M., Flicker S., Gieras A., Westritschnig K., Vrtala S., Balic N., Spitzauer S., Huss-Marp J., Brockow K. (2006). Allergen Cleavage by Effector Cell-derived Proteases Regulates Allergic Inflammation. FASEB J..

[50] Jacquet A. (2011). Interactions of Airway Epithelium with Protease Allergens in the Allergic Response: Interactions of Airway Epithelium with Protease Allergens. Clin. Exp. Allergy.

[51] Yuan J., Yu Z., Wang X., Feng L., Tang J., Li Z. (2026). The Research Progress on the Relationship Between Asthma and Skin Barrier Damage. Br. J. Hosp. Med..

[52] Zimbru E.-L., Zimbru R.-I., Ordodi V.-L., Bojin F.-M., Crîsnic D., Andor M., Mirica S.-N., Huțu I., Tănasie G., Haidar L. (2024). Rosuvastatin Attenuates Vascular Dysfunction Induced by High-Fructose Diets and Allergic Asthma in Rats. Nutrients.

[53] Raby K.L., Michaeloudes C., Tonkin J., Chung K.F., Bhavsar P.K. (2023). Mechanisms of Airway Epithelial Injury and Abnormal Repair in Asthma and COPD. Front. Immunol..

[54] Heijink I.H., Kuchibhotla V.N.S., Roffel M.P., Maes T., Knight D.A., Sayers I., Nawijn M.C. (2020). Epithelial Cell Dysfunction, a Major Driver of Asthma Development. Allergy.

[55] Golebski K., Luiten S., Van Egmond D., De Groot E., Röschmann K.I.L., Fokkens W.J., Van Drunen C.M. (2014). High Degree of Overlap between Responses to a Virus and to the House Dust Mite Allergen in Airway Epithelial Cells. PLoS ONE.

[56] Stanbery A.G., Smita S., Von Moltke J., Tait Wojno E.D., Ziegler S.F. (2022). TSLP, IL-33, and IL-25: Not Just for Allergy and Helminth Infection. J. Allergy Clin. Immunol..

[57] Andor M., Man D.E., Nistor D.C., Buda V., Dragan S. (2024). The Influence of COVID-19 in Glycemic Control: Predictive Value of Inflammation and Metabolic Parameters. Biomedicines.

[58] Hammad H., Chieppa M., Perros F., Willart M.A., Germain R.N., Lambrecht B.N. (2009). House Dust Mite Allergen Induces Asthma via Toll-like Receptor 4 Triggering of Airway Structural Cells. Nat. Med..

[59] Lambrecht B.N., Ahmed E., Hammad H. (2025). The Immunology of Asthma. Nat. Immunol..

[60] Kawasaki T., Kawai T. (2014). Toll-Like Receptor Signaling Pathways. Front. Immunol..

[61] Komlósi Z.I., Van De Veen W., Kovács N., Szűcs G., Sokolowska M., O’Mahony L., Akdis M., Akdis C.A. (2022). Cellular and Molecular Mechanisms of Allergic Asthma. Mol. Asp. Med..

[62] Kawano H., Kayama H., Nakama T., Hashimoto T., Umemoto E., Takeda K. (2016). IL-10-Producing Lung Interstitial Macrophages Prevent Neutrophilic Asthma. Int. Immunol..

[63] Wu Y., Di X., Zhao M., Li H., Bai L., Wang K. (2022). The Role of the NLRP3 Inflammasome in Chronic Inflammation in Asthma and Chronic Obstructive Pulmonary Disease. Immun. Inflamm. Dis..

[64] Kraik K., Tota M., Laska J., Łacwik J., Paździerz Ł., Sędek Ł., Gomułka K. (2024). The Role of Transforming Growth Factor-β (TGF-β) in Asthma and Chronic Obstructive Pulmonary Disease (COPD). Cells.

[65] Kuwabara Y., Kobayashi T., D’Alessandro-Gabazza C.N., Toda M., Yasuma T., Nishihama K., Takeshita A., Fujimoto H., Nagao M., Fujisawa T. (2018). Role of Matrix Metalloproteinase-2 in Eosinophil-Mediated Airway Remodeling. Front. Immunol..

[66] Mukohda M., Ozaki H. (2021). Anti-Inflammatory Mechanisms of the Vascular Smooth Muscle PPARγ. J. Smooth Muscle Res..

[67] Wan R., Srikaram P., Xie S., Chen Q., Hu C., Wan M., Li Y., Gao P. (2024). PPARγ Attenuates Cellular Senescence of Alveolar Macrophages in Asthma-COPD Overlap. Respir. Res..

[68] Stark J.M., Coquet J.M., Tibbitt C.A. (2021). The Role of PPAR-γ in Allergic Disease. Curr. Allergy Asthma Rep..

[69] Zimbru R.-I., Zimbru E.-L., Bojin F.-M., Haidar L., Andor M., Harich O.O., Tănasie G., Tatu C., Mailat D.-E., Zbîrcea I.-M. (2025). Connecting the Dots: How MicroRNAs Link Asthma and Atherosclerosis. Int. J. Mol. Sci..

[70] Carlier F.M., De Fays C., Pilette C. (2021). Epithelial Barrier Dysfunction in Chronic Respiratory Diseases. Front. Physiol..

[71] Calvén J., Ax E., Rådinger M. (2020). The Airway Epithelium—A Central Player in Asthma Pathogenesis. Int. J. Mol. Sci..

[72] Sudharson S., Kalic T., Eckl-Dorna J., Lengger N., Breiteneder H., Hafner C. (2024). Modulation of Bronchial Epithelial Barrier Integrity by Low Molecular Weight Components from Birch Pollen. Int. J. Mol. Sci..

[73] Weng C.-M., Lee M.-J., Chao W., Lin Y.-R., Chou C.-J., Chen M.-C., Chou C.-L., Tsai I.-L., Lin C.-H., Fan Chung K. (2023). Airway Epithelium IgE-FcεRI Cross-Link Induces Epithelial Barrier Disruption in Severe T2-High Asthma. Mucosal Immunol..

[74] Ansaldo E., Farley T.K., Belkaid Y. (2021). Control of Immunity by the Microbiota. Annu. Rev. Immunol..

[75] Kim Y.-C., Sohn K.-H., Kang H.-R. (2024). Gut Microbiota Dysbiosis and Its Impact on Asthma and Other Lung Diseases: Potential Therapeutic Approaches. Korean J. Intern. Med..

[76] Zimbru E.-L., Zimbru R.-I., Bojin F.-M., Chiriac S.D., Haidar L., Andor M., Tănasie G., Tatu C., Georgescu M., Uța C. (2025). Microbiota-Driven Immune Dysregulation Along the Gut–Lung–Vascular Axis in Asthma and Atherosclerosis. Biomedicines.

[77] Yang Y., Jia M., Ou Y., Adcock I.M., Yao X. (2021). Mechanisms and Biomarkers of Airway Epithelial Cell Damage in Asthma: A Review. Clin. Respir. J..

[78] López-Rodríguez J.C., Rodríguez-Coira J., Benedé S., Barbas C., Barber D., Villalba M.T., Escribese M.M., Villaseñor A., Batanero E. (2021). Comparative Metabolomics Analysis of Bronchial Epithelium during Barrier Establishment after Allergen Exposure. Clin. Transl. Allergy.

[79] Agache I., Akdis C.A. (2019). Precision Medicine and Phenotypes, Endotypes, Genotypes, Regiotypes, and Theratypes of Allergic Diseases. J. Clin. Investig..

[80] Albrecht M., Garn H., Buhl T. (2024). Epithelial–Immune Cell Interactions in Allergic Diseases. Eur. J. Immunol..

[81] Aggarwal K., Bansal V., Mahmood R., Kanagala S.G., Jain R. (2023). Asthma and Cardiovascular Diseases: Uncovering Common Ground in Risk Factors and Pathogenesis. Cardiol. Rev..

[82] Tattersall M.C., Guo M., Korcarz C.E., Gepner A.D., Kaufman J.D., Liu K.J., Barr R.G., Donohue K.M., McClelland R.L., Delaney J.A. (2015). Asthma Predicts Cardiovascular Disease Events: The Multi-Ethnic Study of Atherosclerosis. Arterioscler. Thromb. Vasc. Biol..

[83] Gurgone D., McShane L., McSharry C., Guzik T.J., Maffia P. (2020). Cytokines at the Interplay Between Asthma and Atherosclerosis?. Front. Pharmacol..

